# Two Chinese Xia‐Gibbs syndrome patients with partial growth hormone deficiency

**DOI:** 10.1002/mgg3.596

**Published:** 2019-02-06

**Authors:** Xinran Cheng, Fang Tang, Xuyun Hu, Hongduo Li, Mengting Li, Yiyong Fu, Li Yan, Zhonghui Li, Peng Gou, Na Su, Chunzhu Gong, Weilan He, Rong Xiang, Dongmei Bu, Yiping Shen

**Affiliations:** ^1^ Department of Pediatric Endocrinology and Genetic Metabolism Chengdu Women’s and Children’s Central Hospital Chengdu China; ^2^ Genetic and Metabolic Central Laboratory, Birth Defect Prevention Research Institute, Maternal and Child Health Hospital Children’s Hospital of Guangxi Zhuang Autonomous Region Nanning China; ^3^ Neonatal Intensive Care Unit Chengdu Women’s and Children’s Central Hospital Chengdu China; ^4^ Department of Medical Genetics and Molecular Diagnostic Laboratory, Shanghai Children’s Medical Center Shanghai Jiao Tong University School of Medicine Shanghai China; ^5^ Division of Genetics and Genomics Boston Children’s Hospital Boston Massachusetts; ^6^ Department of Neurology Harvard Medical School Boston Massachusetts

**Keywords:** *AHDC1* mutation, next generation sequencing, partial growth hormone deficiency, Xia‐Gibbs syndrome

## Abstract

**Background:**

Heterozygous mutations in the AT‐hook DNA‐binding motif containing one (*AHDC1*, OMIM * 615790) gene cause an autosomal dominant multisystem developmental disorder known as Xia‐Gibbs syndrome (OMIM #615829). Xia‐Gibbs syndrome typically presented with global developmental delay, hypotonia, obstructive sleep apnea, seizures, delayed myelination, micrognathia, and other mild dysmorphic features.

**Methods:**

Description of the clinical materials of two Chinese boys who were diagnosed with Xia‐Gibbs syndrome based on clinical presentations and next generation sequencing. Review of clinical features and *AHDC1* mutations in previously reported Xia‐Gibbs syndrome patients together with our two new patients.

**Results:**

The Xia‐Gibbs syndrome patients exhibited short stature, hypotonia, global developmental delay, speech delay, simian crease, and mild dysmorphic features. Next generation sequencing revealed de novo heterozygous variants in *AHDC1* gene. In addition, laboratory test revealed partial growth hormone deficiency. Both patients underwent growth hormone replacement therapy for 24 and 9 months, respectively, and exhibited good response to the treatment.

**Conclusion:**

This is the first report of Xia‐Gibbs syndrome patients to be treated with growth hormone. Review of previously reported Xia‐Gibbs syndrome patient indicated that short stature is a frequent feature of this condition, but its underlying cause needs to be further investigated.

## 1 INTRODUCTION

Xia‐Gibbs syndrome is a recently described genetic disorder caused by heterozygous mutations in the *AHDC1* gene (Bosch et al., 2016; García‐Acero & Acosta, 2017; Jiang et al., 2018; Xia et al., 2014; Yang et al., 2015) which encodes an AT‐hook DNA‐binding motif‐containing protein 1(Reeves & Nissen, 1990). So far there were been 27 patients reported with Xia‐Gibbs syndrome, most of them were Caucasians (Jiang et al., 2018). They were mainly diagnosed through the identification of de novo variants in *AHDC1* gene by exome sequencing. Patients with Xia‐Gibbs syndrome were typically presented with global developmental delay, hypotonia, obstructive sleep apnea, seizures, delayed myelination, micrognathia, and other mild dysmorphic features (Bosch et al., 2016; García‐Acero & Acosta, 2017; Jiang et al., 2018; Xia et al., 2014; Yang et al., 2015). Yet the whole spectrum of the phenotype is not well established, especially among patients with other ethnicities. Here, we report two Chinese patients with de novo *AHDC1* mutations and the clinical presentations of Xia‐Gibbs syndrome. In addition, both of our patients exhibited short stature and had partial growth hormone deficiency. Furthermore, we evaluated the responses of human recombinant growth hormone replacement treatment. Our analysis also revealed that short stature is a frequent feature of Xia‐Gibbs syndrome, growth hormone may be effective for height improvement. This is also the first report of Chinese Xia‐Gibbs syndrome patients and associated novel pathogenic variant.

## 2 METHODS AND RESULTS

### 2.1 Ethical Statement

This study was approved by the ethics committee of Chengdu Women's and Children's Central Hospital. The study follows the principles outlined in the Helsinki Declaration and the parents of the two patients gave written informed consent for molecular study and publication.

### 2.2 Next generation sequencing

Two patients were enrolled in a study on short stature sequencing program (Hu et al., 2017). Peripheral blood samples were collected after parental consent. DNA was extracted from the peripheral blood leukocytes by using the Gentra Puregene Blood Kit (QIAGEN, Hilden, Germany) according to the manufacturer's protocol. A custom gene panel consisted of 705 short stature‐related genes was designed by NimbleDesign (Roche, Madison, WI, USA). Data analysis was performed as described in a previous article (Hu et al., 2017). Next generation sequencing was done using Hiseq2000 (Illumina, San Diego, USA). Rare variants were evaluated and classified following the ACMG/AMP standards and guidelines (Richards et al., 2015). The GenBank reference sequence and version number for the AHDC1 gene was NM_001029882.3.

### 2.3 Endocrine laboratory tests

Growth hormone stimulation test, IGF‐binding protein 3 test, and insulin‐like growth factor 1 (IGF‐1) test were performed on IMMULITE 2000 (Siemens, LOS Angeles, USA) by enhanced chemiluminescence immunoassay method. Thyroid function test was performed on ADVIA Centaur XP (Siemens, Munich, Germany) by microsome chemiluminescence method. Renal function test, liver function test, and electrolytes test were performed on Hitachi 7600‐020 automatic biochemical analyzer (Hitachi, Tokyo, Japan) by serial detecting method.

## 3 CLINICAL REPORTS

### 3.1 Case 1

**Figure 1:**
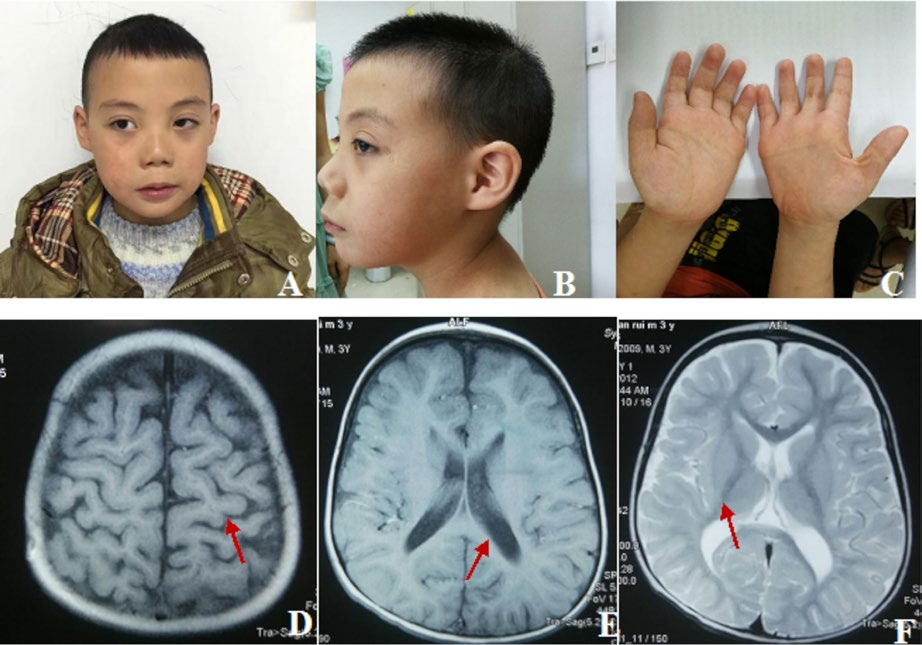
Clinical features of the case 1. (a, b) He had dysmorphic features including hypertelorism, a broad forehead, long philtrum, upslanting palpebral fissures, hypoplastic columella and ala nasi, thin upper lip, high‐arched palate, epicanthic fold, and micrognathia. (c) He had brachydactyly and a simian crease on his right hand. (d–f) Brain magnetic resonance imaging (MRI) scans showed the sulci and the lateral ventricle widened, the white matter volume was less than normal (arrows)

Case 1 was an 8‐year and 2‐month‐old boy. The patient was born at 40 weeks gestation by cesarean section, with a birth weight of 3,200 g (−0.3 *SD*) and a body length of 50 cm (−0.2 *SD*). His parents were healthy and nonconsanguineous. His developmental milestones were delayed, with head control at 10 months, sitting at 12 months, standing at 24 months, walking at 38 months, putting two words together at 24 months. The patient exhibited hypotonia, amblyopia, astigmatism, teeth hypoplasia, and dysmorphic features including hypertelorism, a broad forehead, long philtrum, upslanting palpebral fissures, hypoplastic columella and ala nasi thin upper lip, high‐arched palate, epicanthic fold, and micrognathia. He had brachydactyly and a simian crease on his right hand (Figure 1). Electroencephalogram was abnormal, showing sharp waves and sharp slow complex waves on bilateral forehead and central region. The boy presented to our Endocrinology clinic at the age of 6 years and 2 months with a height of 102 cm (−3.6 *SD*) and a weight of 16.8 kg (−2.2 *SD*) (Z‐scores were calculated based on the China's 2009 urban 0 to 18‐year‐old male height and weight growth reference standards; Li, Ji, Zong, & Zhang, 2009). Urine and plasma amino acid testing revealed hyperlactatemia. Other laboratory test results including thyroid function, serum insulin‐like growth factor I level, Insulin‐like growth factor‐binding protein 3 level, serum glucose, routine urine analysis, routine blood test, renal function test, liver function test, and levels of electrolytes were all within normal ranges. Growth hormone provocative tests revealed that the peak growth hormone levels responding to two provocative tests (clonidine 5 µg/kg, orally, and arginine 0.5 g/kg, intravenously) were 5.60 ng/ml (Table 1). Peak growth hormone levels between 5 and 10 ng/ml on provocative testing are defined as partial growth hormone deficiency according to current guidelines (Grimberg et al., 2016).

**Table 1:** Endocrine evaluation of the patients

Items	Value in patient 1	Value in patient 2	Normal range
Peak GH level responding to arginine 0.5 g/kg, iv (ng/mL)	4.41	7.11	>10
Peak GH level responding to clonidine 10 mg/kg, orally (ng/mL)	5.60	5.25	>10
Serum IGF‐I (ng/mL)	55.70	48.7	52.71–354.71
Serum IGFBP3 (µg/mL)	3.33	2.41	2.65–7.89

Note

IGF‐I, insulin‐like growth factor I; IGFBP3, Insulin‐like growth factor‐binding protein 3; iv, intravenously.

Brain magnetic resonance imaging (MRI) scan showed widened sulci and lateral ventricles and reduced volume of white matter. No signs of hypoplastic corpus callosum, delayed myelination, or simplified gyral pattern (Figure 1). Bone age was delayed and was compatible with that of a 3‐year‐old boy.

**Figure 2:**
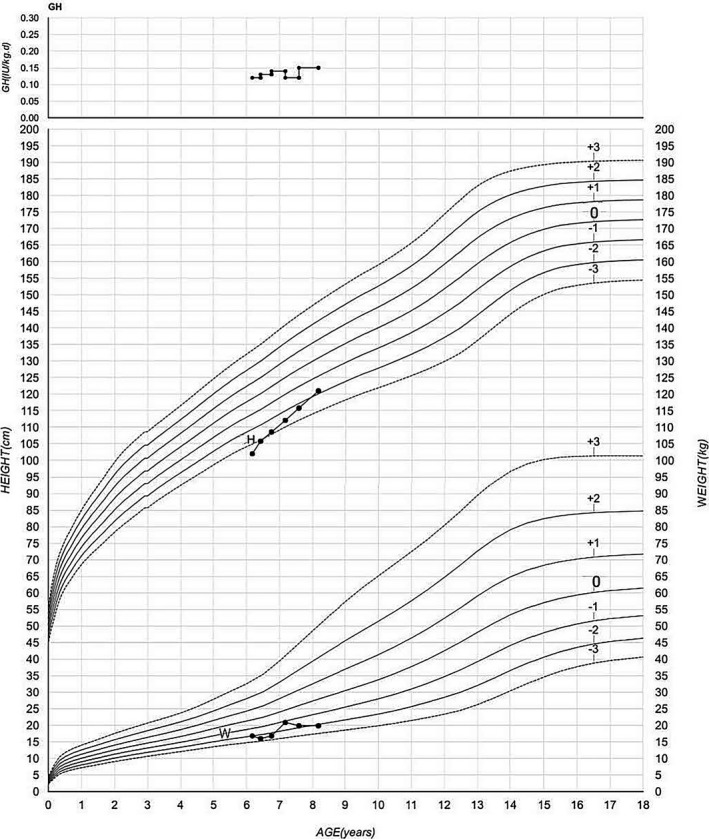
Growth hormone doses and growth chart in case 1 (GH: growth hormone doses; H: height; W: weight)

Due to the partial growth hormone deficiency and short stature, growth hormone replacement therapy at a dose of 0.12–0.15 IU kg^−1^ day^−1^ was initiated. After 2 years of treatment, at the age of 8 years and 2 months, his height was 121.0 cm (−1.9 *SD*) and weight 19.8 kg (−2.4 *SD*). The growth hormone therapy markedly improved the linear growth of the patient with a growth velocity of 9.5 cm/year during the 2 years. Growth hormone doses and growth chart in case 1 are shown in Figure 2. The serum levels of IGF1 increased to 304 ng/ml during the treatment.

### 3.2 Case 2

**Figure 3:**
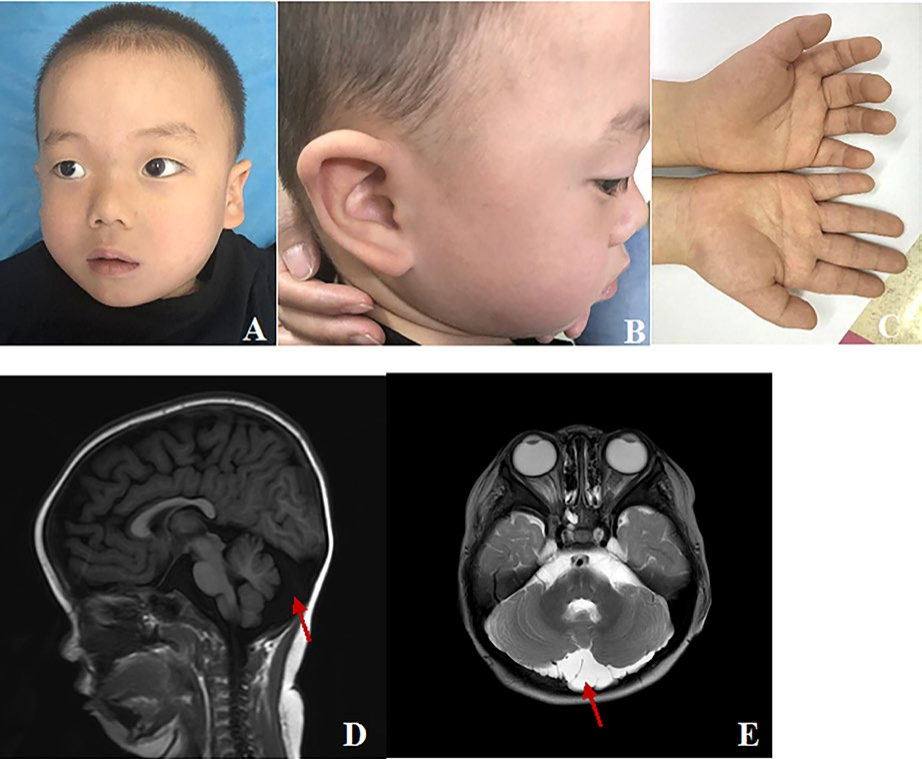
Clinical features of the case 2. (a, b) He had dysmorphic features including a broad forehead, hypertelorism, upslanting palpebral fissures, hypoplastic columella and ala nasi, flat nasal bridge, long philtrum, upturned earlobes, high‐arched palate, micrognathia. (c) He had brachydactyly and simian crease in both palms. (d, e) Brain magnetic resonance imaging (MRI) showed an enlarged cerebellomedullary cistern and arachnoidal cyst

Case 2 was a 4‐year‐old boy. He was the only child of a consanguineous parents. Pregnancy and delivery were uneventful. He was born with a birth weight of 2,900 g (−1 *SD*) and a birth length of 50 cm (−0.2 *SD*). He held head at 7 months, sat at 13 months, stood at 18 months, walked at 24 months, put two words together at 20 months, spoke in full sentences at 3 years. He exhibited hypotonia. His dysmorphic features include a broad forehead, hypertelorism, upslanting palpebral fissures, hypoplastic columella and ala nasi, flat nasal bridge, long philtrum, upturned earlobes, high‐arched palate, micrognathia. He had brachydactyly and simian crease in both palms (Figure 3). The boy was presented to our Endocrinology clinic at the age of 3 years and 6 months. At that time, his height was 90 cm (−2.9 *SD*), and weight 15.5 kg (−0.1 *SD*). Laboratory test results including the thyroid function, serum glucose, routine urine analysis, routine blood test, renal function test, liver function test, and the levels of electrolytes were within normal ranges. Growth hormone provocative test also revealed a partial growth hormone deficiency (7.11 ng/ml). The serum insulin‐like growth factor I level was low (48.7 ng/ml) and insulin‐like growth factor‐binding protein 3 was low (2.41ug/mL) (Table 1). Brain magnetic resonance imaging (MRI) showed an enlarged cerebellomedullary cistern and arachnoidal cyst. (Figure 3). Bone age was delayed and was compatible with that of a 1.5 years old boy.

**Figure 4:**
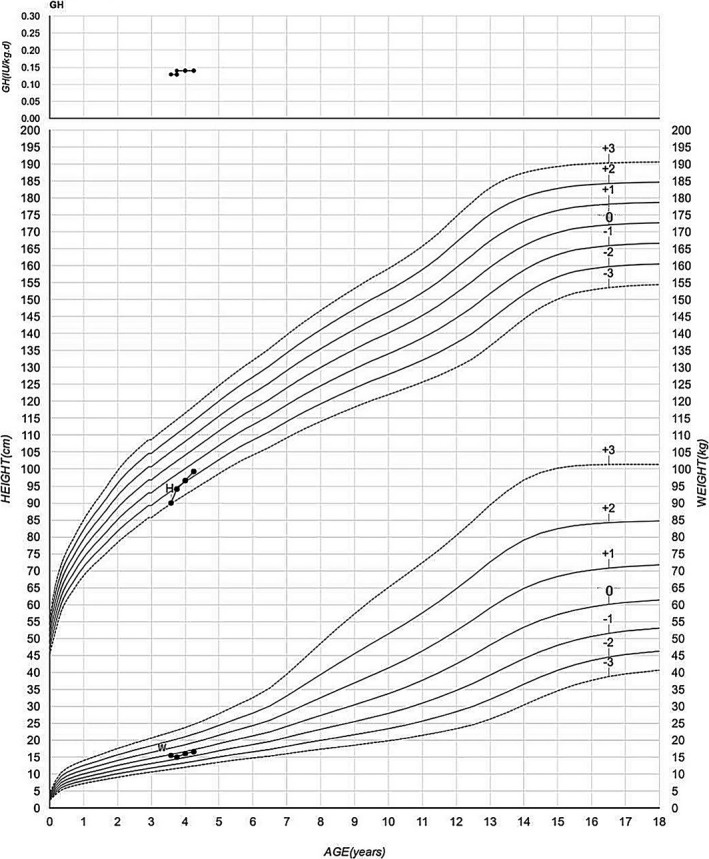
Growth hormone doses and growth chart in case 2 (GH: growth hormone doses; H: height; W: weight)

The patient also underwent growth hormone replacement therapy at a dose of 0.13–0.15 IU kg^−1^ day^−1^. After being treated for 9 months, at the age of 4 years and 3 months, his height was 99.3 cm (−1.7 *SD*) and weight 16.6 kg (−0.4 *SD*). The growth velocity during 9 months is 12.4 cm/year. Growth hormone doses and growth chart in case 2 are shown in Figure 4. The serum levels of IGF1 increased to 209 ng/ml during the treatment.

### 3.3 Identification of *AHDC1* mutations

We identified a de novo *AHDC1* frameshift mutation c.2889_2892delTGCC (p.A964fs*177) in patient 1 and a de novo *AHDC1* frameshift mutation c.2373_2374delTG (p.Cys791Trpfs*57) in patient 2. Both mutations were confirmed by Sanger sequencing and were absent in both parental samples. The variant in patient 1 is novel, the variant in patient 2 is a recurrent variant (Jiang et al., 2018; Xia et al., 2014; Yang et al., 2015). Both variants are classified as pathogenic flowing the ACMG/AMP sequence variant classification guideline.

## 4 DISCUSSION

De novo loss‐of‐function mutations in *AHDC1 *gene cause Xia‐Gibbs syndrome, a recently described genetic disorder characterized by failure to thrive, hypotonia, global developmental delay, and mild dysmorphic features including hypertelorism, a broad forehead, flat nasal bridge, and thin upper lip. Less than 30 patients had been reported world‐wide. Here, we described two unrelated Chinese male patients who carried de novo frameshift mutations, c.2889_2892delTGCC (p.A964fs*177) and c.2373_2374delTG (p.Cys791Trpfs*57) in *AHDC1* gene. The first mutation is novel and the second one is a recurrent pathogenic variant that had been reported in four Xia‐Gibbs patients (Jiang et al., 2018; Xia et al., 2014; Yang et al., 2015). The evaluation of Chinese patients with Xia‐Gibbs syndrome helped to expand both clinical and molecular spectra of this condition.

The clinical presentation of Chinese Xia‐Gibbs patient is largely consistent with what had been previously reported. Patient 1 exhibited failure to thrive after birth, both height and weight were significantly below −2 *SD*. Patient 2 only exhibited postnatal growth retardation. Both exhibited hypotonia, delayed developmental milestones, and language impairment. Shared dysmorphic features included hypertelorism, a broad forehead, upslanting palpebral fissures, hypoplastic columella and ala nasi, long philtrum, high‐arched palate, and micrognathia. Nevertheless, we did not observe the following features that were described before in Xia‐Gibbs syndrome patients: low‐set ears and protuberant ears. We observed upturned earlobe in only patient 2. Obstructive sleep apnea was denied by both patients. Interestingly, we observed brachydactyly in both patients, this is a feature that had not been described before.

Most importantly, we noticed that both patients exhibited partial growth hormone deficiency. This clinical feature had not been previously described. We reviewed all previously reported Xia‐Gibbs syndrome cases (Table 2, including two cases in this report). There are 29 patients from unrelated families ranged in age from 18 months to 22 years with an average age of 8.87 years old, including 15 males and 14 females. All variants are truncating mutations. Three recurring mutations were reported, which include five patients share mutation p.Cys791Trpfs*57 (c.2373_2374delTG), two patients share mutation p.Arg925* (c.2773C>T), and two patients share mutation p.Gln970*(c.2908C>T). We noticed that short stature is a common feature of Xia‐Gibbs syndrome. Fifteen of the 26 patients (57.7%) with height information had short stature. Twenty‐five ofthe 28 patients (89.3%) with information had hypotonia, nine of the 24 patients (37.5%) had sleep apnea, 20 of the 29 patients (69.0%) had brain abnormality. All patients had motor delay and speech delay.

**Table 2:** *AHDC1* variants in patients with Xia‐Gibbs syndrome

	Nucleotide change	Protein change	Gender	Age	Short stature (15/26, 57.7%)	Motor delay (28/28, 100%)	Speech delay (28/28, 100%)	Hypotonia (25/28, 89.3%)	Sleep apnea (9/24, 37.5%)	Brain abnormality (19/29, 65.5%)	Reference
1.	C.784C>T	p.Gln262*	F	6 yr	+	+	+	+	−	−	Jiang et al. (2018)
2.	c.1122dupC	p.G375Rfs* 3	F	4 yr	−	+	+	+	unknown	+	Yang et al. (2015)
3.	c.1402dupT	p.Cys468Leufs*49	F	22 yr	unknown	+	+	unknown	unknown	+	Bosch et al. (2016)
4.	c.1480A>T	p.K494X	F	16 yr	+	+	+	−	unknown	+	Yang et al. (2015)
5.	c.1881delG	p.Q627Hfs*105	M	5 yr	−	unknown	unknown	+	unknown	−	Yang et al. (2015)
6.	c.1945delG	p.Ala649Profs*83	M	2 yr	+	+	+	+	+	−	Yang et al. (2015)
7.	c.2030–2030del	p.G677Afs*55	F	8 yr	unknown	+	+	+	+	+	García‐Acero and Acosta (2017)
8.	c.2062C>T	p.Arg688*	F	20 yr	−	+	+	−	−	+	Jiang et al. (2018)
9.	c.2229delG	p.Ser744Profs*188	M	13 yr	+	+	+	+	+	−	Jiang et al. (2018)
10.	c.2373_2374delTG	p.Cys791Trpfs*57	F	18mo	+	+	+	+	+	+	Xia et al. (2014)
			M	8 yr	−	+	+	+	+	+	Xia et al. (2014)
			M	5 yr	−	+	+	+	unknown	+	Yang et al. (2015)
			M	21 yr	+	+	+	+	+	+	Jiang et al. (2018)
			M	4 yr	+	+	+	+	−	+	Present case
11.	c.2415delG	p.Leu806Trpfs*126	F	6 yr	−	−	+	+	−	−	Jiang et al. (2018)
12.	c.2520delT	p.Arg841Alafs*91	M	9 yr	−	+	+	+	−	+	Jiang et al. (2018)
13.	c.2529_2545del17	p.D845Rfs*40	F	7 yr	−	+	+	+	−	+	Yang et al. (2015)
14.	c.2547delC	p.Ser850Profs*82)	M	11 yr	+	+	+	+	−	+	Xia et al. (2014)
15.	c.2644C>T	p.Gln882*	M	10 yr	+	+	+	+	−	+	Jiang et al. (2018)
16.	c.2691delA	p.Val898Trpfs*34	F	4 yr	+	+	+	+	−	+	Jiang et al. (2018)
17.	c.2773C>T	p.Arg925*	F	6 yr	−	+	+	+	−	+	Jiang et al. (2018)
			M	3 yr	+	+	+	+	+	−	Jiang et al. (2018)
18.	c.2898delC	p.Tyr967Thrfs*175	F	4 yr	+	+	+	+	+	+	Xia et al. (2014)
19.	c.2908C>T	p.Gln970*	F	11 yr	unknown	+	+	+	−	−	Jiang et al. (2018)
			F	6 yr	−	+	+	+	−	−	Jiang et al. (2018)
20.	c.3773C>G	p.Ser1258*	M	2 yr	+	+	+	−	+	+	Jiang et al. (2018)
21.	c.3809delA	p.Gln1270Argfs*75	M	9 yr	−	+	+	+	unknown	+	Yang et al. (2015)
22.	c.3989C>A	p.Ser1330*	M	17 yr	+	+	+	+	−	−	Jiang et al. (2018)
23.	c.2889_2892delTGCC	p.A964fs*177	M	8 yr	+	+	+	+	−	+	Present case

It is well known that random serum growth hormone measurement is not useful for the diagnosis of growth hormone deficiency. We performed repeated growth hormone provocative tests in both Chinese patients and detected peak growth hormone levels of 5.6 ng/ml in patient 1 and 7.11 ng/ml in patient 2. Current guidelines (Grimberg et al., 2016) indicated that a peak growth hormone level of >10 ng/ml on provocative testing indicates nongrowth hormone deficiency, and a peak growth hormone level of either <3 or <5 ng/ml is defined as severe growth hormone deficiency. Partial growth hormone deficiency is defined as the peak growth hormone levels between 5 and 10 ng/ml. Both our patients belong to the category of partial growth hormone deficiency. Growth hormone replacement therapy was initiated before the molecular diagnosis of Xia‐Gibbs syndrome. Remarkably, we noticed excellent response to growth hormone treatment in both patients with growth velocity at 9.5 cm/year at age of 8 and >10 cm/year at age of 4, respectively. We did not notice any adverse effects in the duration of the treatment. It is not known if partial growth hormone deficiency is a consistent feature of Xia‐Gibbs syndrome. This is the first report demonstrating the effectiveness of growth hormone replacement therapy for Xia‐Gibbs syndrome patients. The long‐term benefits of growth hormone replacement therapy for Xia‐Gibbs patients are yet to be evaluated. More data are needed to guide the clinical utilization of growth hormone for Xia‐Gibbs syndrome patients.

## CONFLICT OF INTEREST

All authors declare that there is no conflict of interest.
